# Chromatin signature discovery via histone modification profile alignments

**DOI:** 10.1093/nar/gks848

**Published:** 2012-09-18

**Authors:** Jianrong Wang, Victoria V. Lunyak, I. King Jordan

**Affiliations:** ^1^School of Biology, Georgia Institute of Technology, Atlanta, GA 30332, USA, ^2^Buck Institute for Age Research, 8001 Redwood Blvd., Novato, CA 94945, USA and ^3^PanAmerican Bioinformatics Institute, Santa Marta, Magdalena, Colombia

## Abstract

We report on the development of an unsupervised algorithm for the genome-wide discovery and analysis of chromatin signatures. Our Chromatin-profile Alignment followed by Tree-clustering algorithm (ChAT) employs dynamic programming of combinatorial histone modification profiles to identify locally similar chromatin sub-regions and provides complementary utility with respect to existing methods. We applied ChAT to genomic maps of 39 histone modifications in human CD4^+^ T cells to identify both known and novel chromatin signatures. ChAT was able to detect chromatin signatures previously associated with transcription start sites and enhancers as well as novel signatures associated with a variety of regulatory elements. Promoter-associated signatures discovered with ChAT indicate that complex chromatin signatures, made up of numerous co-located histone modifications, facilitate cell-type specific gene expression. The discovery of novel L1 retrotransposon-associated bivalent chromatin signatures suggests that these elements influence the mono-allelic expression of human genes by shaping the chromatin environment of imprinted genomic regions. Analysis of long gene-associated chromatin signatures point to a role for the H4K20me1 and H3K79me3 histone modifications in transcriptional pause release. The novel chromatin signatures and functional associations uncovered by ChAT underscore the ability of the algorithm to yield novel insight on chromatin-based regulatory mechanisms.

## INTRODUCTION

Histone proteins are subject to a variety of covalent modifications, including methylation, acetylation, phosphorylation and ubiquitylation. The identities and locations of these histone modifications have profound effects on the structure and regulatory properties of eukaryotic chromatin (1). Indeed, over the last several years specific genomic regulatory elements, such as promoters, enhancers and boundary elements have been associated with distinct combinatorial patterns of histone modifications (2–12). The discovery and characterization of such combinatorial histone modification patterns, or chromatin signatures as they are often referred to, can provide valuable information with respect to the location and activity of cell type and developmentally specific genomic regulatory features (13–21).

Next-generation sequencing-based technologies, chromatin immunoprecipitation followed by high throughput sequencing (ChIP-seq) in particular, provide an opportunity for the systematic analysis of combinatorial histone modification patterns genome-wide (22,23). Computationally, the inference of combinatorial histone modification signatures is a pattern recognition problem in high-dimensional space. There are currently two classes of computational approaches designed for this purpose: supervised and unsupervised methods. Supervised methods identify histone modification signatures characteristic of a pre-defined set of known genomic features, e.g. promoters or enhancers (6,7,21,24). Regulatory element characteristic combinatorial modification patterns identified in this way can then be used to query the genome to identify the locations of additional regulatory elements of the same kind. The use of supervised methods in this way was critically important for the discovery that specific genomic regulatory elements bear distinct chromatin signatures. However, supervised methods are unsuited for the discovery of novel histone modification patterns that may be associated with as yet unknown regulatory activities. Unsupervised methods do not rely on training data sets derived from previously annotated features, and as such they have the potential to discover the kinds of unknown chromatin signatures that characterize novel regulatory elements. Here, we are more interested in the unsupervised approach to the analysis of chromatin given the potential this approach holds for novel discoveries.

There are a number of available unsupervised algorithms for the analysis of histone modification patterns. The program ChromaSig utilizes probabilistic profiles that are characteristic of specific histone modification patterns (25,26). The CoSBI algorithm applies a biclustering method to search for regions with common histone modification patterns (27). Hidden Markov Model (HMM) based methods are widely used to segment eukaryotic genomes into various combinatorial chromatin states with distinct histone modification profiles (15,28,29). The most recently developed method of this kind, Segway, employs Dynamic Bayesian Networks to achieve greater precision for the detection of known regulatory elements along with superior accommodation of missing data (30).

We have developed an unsupervised algorithm for analysis of combinatorial histone modification patterns that extends the capabilities of existing methods in a number of ways. First, our method does not apply any restriction to the size of co-located histone modification patterns. Second, our method does not utilize any motif seed to initialize the subsequent inference of histone modification patterns. Third, our method is capable of detecting histone modification patterns with multiple modes, e.g. co-located signatures made up of constituent individual modifications that are spatially shifted with respect to one another. Fourth, our method is capable of detecting co-located signatures composed of alternating segments with conserved and variant combinatorial patterns. Fifth, our method discriminates between chromatin signatures composed of the same histone modifications but with different shapes. Sixth, our method provides an inherent statistical criterion that allows related chromatin signatures to be classified into distinct groups, and thereby delineates the total number of patterns observed in any data set. The first four features described earlier distinguish our method from the ChromaSig and CoSBI programs. The fifth feature provides added utility beyond what is available for the HMM and Segway methods, and the sixth statistical feature is uniquely implemented in our approach.

We call our method ChAT, for *Ch*romatin-profile *A*lignment followed by *T*ree-clustering, and we applied this approach to the genome-wide analysis of 39 histone modifications characterized by ChIP-seq analysis of human CD4^+ ^T cells (3,11). Application of ChAT on this data set resulted in the discovery of chromatin signatures previously shown to be characteristic of specific genomic regulatory elements along with a number of novel chromatin signatures and features that point to as yet unexplored chromatin-related regulatory mechanisms. We report these discoveries in light of the design and implementation of the ChAT algorithm, with an emphasis on comparison with existing methods. The ability of the ChAT algorithm to discern combinatorial histone modification patterns previously observed to be associated with known regulatory elements serves as proof of its utility for the discovery of functionally relevant chromatin signatures. The characterization of previously undiscovered chromatin signatures and functional associations with ChAT supports the potential utility of the algorithm to yield novel biological insight.

## MATERIALS AND METHODS

### General scheme of the ChAT algorithm

**Figure 1.**
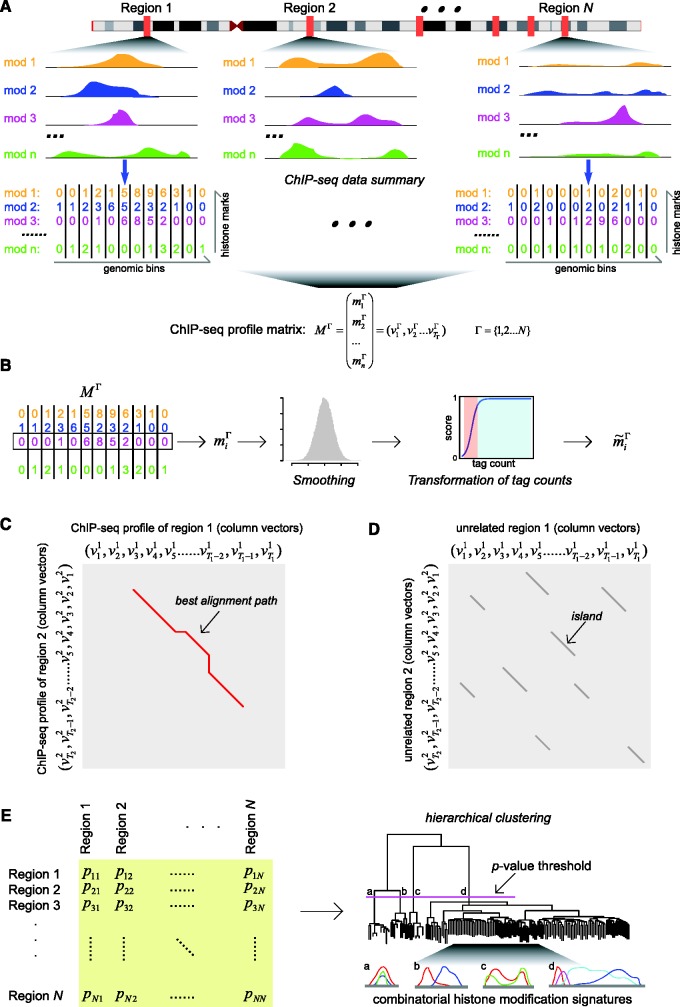
Scheme of the ChAT algorithm. (**A**) For a series of 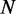 genomic regions, combinatorial histone modification distributions are represented by ChIP-seq profile matrices. Each genomic region under consideration is divided into 200 bp non-overlapping bins and each bin is associated with a column vector (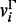) summarizing the ChIP-seq tag counts for 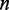 different histone modifications. The contiguous landscape of each individual histone modification along the genomic region is represented by the corresponding row vector (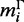). (**B**) Histone modification ChIP-seq tag counts are smoothed and transformed to produce normalized scores. (**C**) Dynamic programming is used to identify sub-regions with similar chromatin signatures. For each pair of genomic regions, a local dynamic programming algorithm is used to compare column vectors 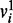 vs. 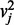 (i.e. the combinatorial histone modification signatures of individual genomic bins), and the best alignment path (red) is identified. (**D**) Pairwise *P*-values are computed based on a null distribution of high-scoring chromatin segment pairs (islands) found between unrelated genomic regions. Dynamic programming is used to identify high-scoring islands (grey lines), and the score distributions of the islands are used to estimate the parameters of extreme-value distributions for *P*-value calculation. (**E**) Pairwise *P*-values are organized into a distance matrix that is used for hierarchical clustering of similar chromatin sub-regions. The resulting tree of chromatin signatures can be partitioned using an explicit *P*-value threshold (purple line) to identify groups of related signatures.

The ChAT algorithm analyzes genome-wide histone modification data sets produced via ChIP-seq to characterize distinct chromatin signatures. ChAT is an unsupervised algorithm; its use does not require any training set based on pre-defined genomic annotations such as the locations of promoters, enhancers or transcription factor binding sites. There are three major steps in the ChAT algorithm: (i) ChIP-seq data transformation, (ii) dynamic programming on histone modification profiles and (iii) hierarchical clustering of genomic regions that correspond to related chromatin signatures (Figure 1).

### ChIP-seq data transformation

The genome is divided into 200 bp non-overlapping bins, and for each bin arrays of ChIP-seq signals (i.e. tag counts) for all histone modifications in the data set are computed. In this way, combinatorial histone modification profiles are represented as a matrix 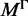, where 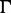 is the index of the genomic regions ranging from 1 to 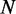 (assuming there are totally 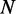 genomic regions under consideration). For each region, the number of columns (i.e. the number of bins within that region) is denoted as 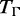 and the number of rows (i.e. the number of histone modifications) is denoted as 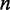. The column vectors correspond to combinatorial histone modification tag counts within individual genomic bins and the row vectors correspond to the contiguous genomic landscape of individual histone modifications (Figure 1A). Then for each individual histone modification (i.e. each row vector), the tag counts are smoothed using a Gaussian kernel (with variance set to 1) to remove noise resulting from spurious tag counts in the ChIP-seq experiments (Figure 1B). The resulting smoothed ChIP-seq tag counts for each histone modification are transformed to a score between 0 and 1 for all subsequent analysis (Figure 1B).

The transformation is: 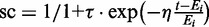, where “sc” is the transformed score and 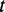 is the smoothed tag count. 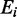 is the genomic median of tag counts of histone modification 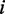. Thus, the transformation is dependent on the genomic tag count distributions for each specific histone modification. For the analysis reported here, 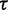 is set as 9 and 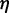 is set as 2.19. In this way, the median tag count is transformed to the score of 0.1 and a tag count twice as big as median is transformed to the score of 0.5. The transformation is performed for two reasons. First, the vast majority of bin tag counts for each histone modification are very small (e.g. 1 or 2 tags), and the transformation allows such regions to be effectively excluded from subsequent analysis. Second, large differences between high bin tag count values (e.g. 100 versus. 150 tags) can bias subsequent alignment steps, and the transformation allows the magnitude of such differences to be dampened.

Having quantified and transformed ChIP-seq histone modification tag count signals in this way, the algorithm then divides the genome into discrete genomic regions (Figure 1A) by delineating contiguous regions that contain high ChIP-seq signals for at least one histone modification from intervening regions that do not contain any such signal. The intervening genomic regions that do not contain any high ChIP-seq signal are excluded from subsequent analysis, and the contiguous genomic regions with high ChIP-seq signal are taken as discrete units for subsequent alignment and chromatin signature analysis. To do this, consecutive genomic bins with high ChIP-seq signals (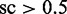) are first merged into a single region, and regions which are close to each other (<1 kb) are further merged together. Importantly, at this step no size threshold or limit for contiguous regions is used. This allows the algorithm to characterize chromatin signatures across a wide range of genomic sizes. In addition, consecutive bins do not need to be enriched with the same histone modification to be merged. This allows the algorithm to characterize chromatin signatures with spatially shifted patterns of individual histone modifications.

To make the algorithm more computationally efficient, individual genomic regions with similar histone modification profiles are grouped together prior to profile alignment with dynamic programming. This grouping is achieved via a simple two-step clustering procedure. First, genomic regions are checked for presence or absence of a set of user-defined histone modifications (e.g. H3K4me3, H3K27ac, H3K27me3 and H3K36me3), and regions are grouped together if they contain the same sets of these modifications. This step reflects the fact that regions which differ with respect to the presence/absence of critical user-defined histone modifications are unlikely to have similar chromatin signatures. Second, genomic regions are further grouped into three size categories: small (≤5 kb), medium (>5 kb and <10 kb) and large (≥10 kb). This initial grouping greatly reduces the number of pairwise profile alignments needed to be performed. It also allows for intelligent user input with respect to the coherence of functionally related (e.g. active vs. repressive) histone modifications.

### Dynamic programming on histone modification profiles

For every pair of genomic regions within the same group, local pairwise alignment of transformed histone modification profile matrices is performed using dynamic programming. The dynamic programming approach entails a number of advantages: it does not require any prior chromatin signature motif seed, it guarantees optimal local alignments that can include gaps, it allows for the discovery of chromatin signatures of vastly different sizes, and it allows for the calculation of *P* values that quantitatively measure chromatin signature similarities between genomic regions.

To perform dynamic programming, the transformed histone modification profile matrix of each discrete genomic region is considered as a string of column vectors and a modified cosine similarity is used as the score to measure the similarity between each pair of column vectors (Figure 1C). For example, the column vector for bin 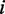 of the first region (region 1) of a pair under comparison is denoted as 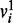. Each entry of this column vector corresponds to the transformed score for the level of a specific histone modification, e.g. 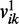 is the value for the 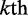 histone modification in bin 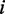. Similarly, the vector for bin 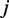 of the second region (region 2) of a pair under comparison is denoted as 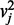 and 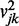 is the value for the 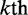 histone modification in bin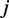. The raw score for the similarity between 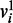 and 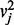 is calculated as: 
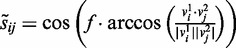
.

The factor 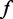 is an amplification factor (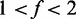) that enlarges the angle between 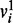 and 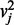. The value of 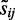 is more likely to be negative with higher values of 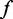 and accordingly the two bins will have lower probability of being aligned. Thus, increasing the value of 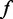 will cause the alignment to be more stringent. Here, 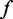 is set to 2 for small-sized region comparisons in order to focus on highly similar sub-regions and is set to 1.5 for medium and large size comparisons.

The raw score is further multiplied by a weight factor to calculate the final score for 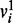 and 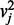. The final score is 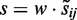 and the weight factor is related to 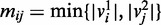. The relation between 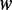 and 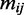 is 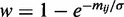. Thus, vectors with small norms are given small weight; the rationale being that vectors with small norms have low levels of ChIP-seq signals and therefore should contribute less to the final signatures even if they are very similar with each other. 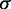 is used to control the stringency of the weight factor. Larger values of 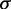 result in smaller weights, and accordingly only genomic regions with abundant ChIP-seq signals will be aligned. Here, 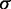 is set as 0.3.

The gap penalty is designed to be proportional to the vector norm. For example, the gap penalty of aligning 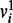 to a gap is 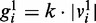. The gap penalty scheme is designed such that it highly penalizes the alignment of vectors with large norms (i.e. high levels of ChIP-seq signals) to gaps. The parameter 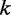 is used to control the stringency of the alignment, and it is designed to be larger for small size region comparisons and smaller for medium and large size comparisons. The introduction of gaps using this scheme enables the discovery of multi-modal chromatin signatures, particularly for large-sized signatures that often contain combinations of conserved and variant segments.

Having parameterized the dynamic programming algorithm in this way, it is then used to search for the most similar sub-regions between pairs of transformed histone modification matrices representing discrete genomic regions. Each entry of the alignment matrix for dynamic programming is as follows:


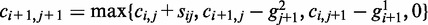
, and 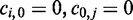. Each pair of regions is compared twice: in the same and in the opposite orientations. In this way, sub-regions with the highest combinatorial histone modification profile similarities will be found.

*P*-values are calculated to quantify the similarities between genomic sub-regions aligned in this way (Figure 1D). To do this, the algorithm employs the island method, based on the extreme value distribution of high-scoring segment pairs, originally developed for DNA sequence comparisons (31). This method creates a null distribution of random similarity scores, against which the observed similarity scores can be compared to compute *p*-values for aligned pairs of sub-regions. To create the null distribution of random similarity scores, pairs of unrelated genomic regions are randomly sampled from the entire set of regions under consideration. Then for each pair of unrelated regions, dynamic programming with the same parameter settings is applied and all high-scoring islands of similarity, with scores above a threshold 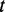, are retained. Using those high-scoring islands, the parameters 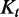 and 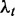 for the extreme value distribution are estimated as suggested by Altschul *et al.* (32), and finally the *P*-value is calculated as: 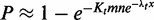.

### Hierarchical clustering of related chromatin signatures

All *P*-values for pairwise profile alignments are organized into a pairwise distance matrix, and hierarchical clustering is applied on this matrix (Figure 1E). In this way, sub-regions with the same combinatorial histone modification signatures will be grouped together and the branch lengths among them in the hierarchical tree will be shorter. Furthermore, because *P*-values are used as pairwise distances, the branch lengths can be viewed as approximate *P*-values among sub-groups or clusters. Then, for a given *P*-value threshold (e.g. 0.05), the hierarchical tree divided by this threshold will yield clusters of related sub-regions at user-defined levels of statistical confidence (Figure 1E). Cluster-characteristic combinatorial histone modification signatures can then be derived.

### Chromatin signature feature enrichment analysis

Chromatin signatures discovered via the application of ChAT to genome-wide histone modification data sets are evaluated for the enrichment over annotated genomic features (e.g. promoters and enhancers) using a fold enrichment (FE) criterion: 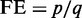, where 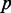 is the fraction of the patterns overlapping with specific genomic features, and 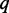 is the fraction of the specific genomic feature in the genome. Here, an FE threshold of 3 was taken to indicate that a given chromatin signature is enriched over a particular genomic feature. The features analysed include transcriptional start site (TSS) (8 kb sequences centered on the transcription start sites of Refseq gene models), transcriptional termination sites (TTS) (8 kb sequences centered on the transcription termination sites of Refseq gene models), enhancers (CD4^+^ T-cell specific p300 binding sites) (33) and CD4^+^ T-cell DNase I hypersensitive sites (34).

## RESULTS AND DISCUSSION

### The ChAT algorithm for chromatin signature discovery

As its name implies, the ChAT algorithm analyzes genome-wide maps of histone modifications characterized by ChIP-seq studies via a process of Chromatin-profile Alignment followed by Tree-clustering. To do this, chromatin profiles are represented as numeric matrices with transformed scores for each histone modification along the genomic sequence (Figure 1A and B). Alignment of these profiles is performed using an implementation of the local dynamic programming algorithm, which allows for the detection of genomic sub-regions with shared chromatin profiles (Figure 1C). Dynamic programming also allows for the introduction of gaps in the chromatin profile alignments. Gaps are critical because they allow the algorithm to extend beyond regions with variant (or diffuse) chromatin enrichment signatures, and in so doing facilitate the discovery of chromatin signatures that span long genomic regions as well as those with complex multi-modal patterns of histone modification enrichment. For each resulting pairwise chromatin profile alignment, an approximate *P* value is calculated (Figure 1D), and hierarchical clustering is then applied on these pairwise values to organize genomic regions into related groups of chromatin signatures (Figure 1E). The use of *P* values for clustering allows for an inherent statistical criterion by which the hierarchical tree can be divided into groups of coherent chromatin signatures. Software to run the ChAT algorithm is freely available at http://jordan.biology.gatech.edu/page/software/ChAT. Detailed instructions for running the ChAT software can be found on the webpage and in Supplementary File S1.

ChAT is distinguished from existing methods for the analysis of chromatin signatures in a number of ways. The collection of algorithmic features that characterize ChAT are compared with their presence among existing methods ChromaSig, CoSBI, ChromHMM and Segway in Supplementary Table S1. ChAT is unique among these methods in that it does not have any size restriction, it does not use chromatin signature motif seeds, it can discover signatures with multi-modes and distinct shapes and it possesses an intrinsic statistical criterion.

ChAT performs a mode of chromatin signature analysis that differs from the analyses performed by ChromHMM and Segway, both of which segment the entire genome into adjacent distinct chromatin states. ChAT searches for recurrent chromatin signatures present at different locations across the genome, similar to the analyses performed by ChromaSig and CoSBI. The similarity among ChAT and these latter two methods allows for a qualitative comparison of their performance on the CD4^+^ T-cell histone modification data sets analyzed here. The methods perform similarly for the discovery of small mono-modal signatures (Supplementary Figure S1A), but differ substantially when it comes to the discovery of more complex chromatin signatures (Supplementary Figure S1B–S1D). For example, ChAT is able to distinguish bi-modal from mono-modal signatures, it is able to distinguish signatures that are made up of the same constituent modifications but have different shapes, and it is able to discern highly complex large signatures. Examples of the ability of ChAT to discover these kinds of complex chromatin patterns are described in more detail later in the context of specific biological features that the algorithm helps to uncover.

### Application of ChAT to CD4^+^ T-cell chromatin

We applied the ChAT algorithm to the analysis of genome-wide maps of 39 histone modifications characterized using ChIP-seq on human CD4^+^ T cells (3,11) in an attempt to discover all discernible histone modification patterns. ChAT was run using the parameter values described in the Materials and Methods section, and a *P*-value threshold of 0.05 was used to partition the resulting hierarchical trees of patterns to explicitly delineate individual chromatin signatures. As stated previously, application of ChAT to ChIP-seq histone modification data sets does not require any restriction on the size of potential chromatin signatures or the use of motif seeds to initialize the search.

ChAT identified a total of 206 distinct combinatorial histone modification patterns genome-wide, which were subsequently grouped into small- (144), medium- (35) and large-sized (27) categories as explained in the Materials and Methods. Overall, the features of these observed chromatin signatures are consistent with the intended design of the algorithm and point to the additional utility provided by its use. For instance, we detected a number of large-sized patterns, ranging from 10 to 100 kb, which demonstrate the utility of allowing alternating conserved and variant segments in the detection scheme. We also find a number of signatures with multiple modes of histone modifications as well as spatially shifted patterns for individual constituent modifications. Combinatorial patterns that bear the same individual histone modifications with different relative profile shapes are recognized as distinct chromatin signatures.

Inspection of the small-sized patterns revealed that a substantial fraction of these signatures are associated with known regulatory features, such as TSS, TTS and p300 binding sites (Supplementary Table S2). A total of 41.7% of the small-sized patterns are enriched with DNase I hypersensitive sites, using a FE threshold of 3 (FE > 3), implying that they are located in open chromatin and possibly co-located with individual regulatory elements. In the following sections, we describe a number of the chromatin signatures discovered by ChAT, with an emphasis on the characterization of known regulatory features, which serve as a kind of positive control for the approach, along with descriptions of previously uncharacterized patterns that underscore the ability of the algorithm to facilitate novel discoveries.

### TSS-associated chromatin signatures

**Figure 2.**
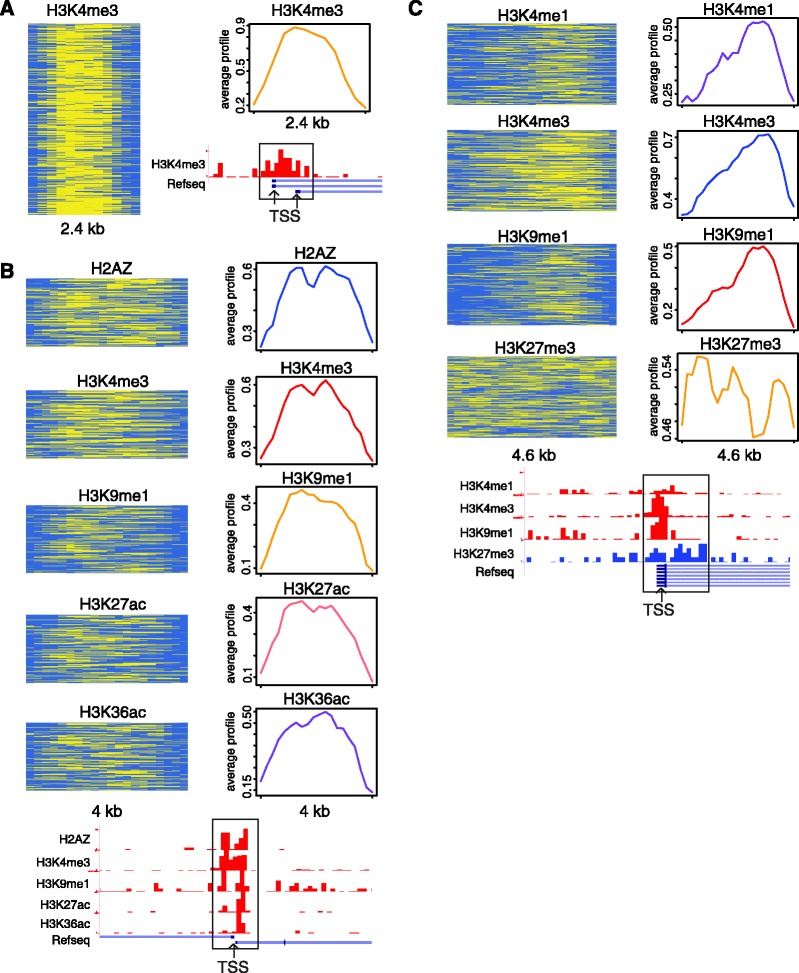
TSS-associated chromatin signatures. (**A**) A TSS-associated signature based on enrichment of H3K4me3 is represented as a heatmap (yellow, high; blue, low levels of modification) and an enrichment profile showing the average modification scores across the signature. H3K4me3 tag counts (red) are shown for an instance of this signature at a human promoter locus. (**B**) A TSS-associated signature composed of five active histone modifications along with an example of this pattern seen at a divergent promoter locus. (**C**) A bivalent TSS-associated signature with three active modifications and one repressive modification (H3K27me3). Distributions of the active (red) and repressive (blue) histone modification tag counts are shown for a single promoter locus.

Because chromatin signatures around active TSS have been previously well-characterized (3,7), we searched for ChAT identified chromatin signatures that are co-located with annotated TSS in an attempt to evaluate the performance of the algorithm. There are 36 small-sized signatures that were found to be enriched at TSS (Supplementary Table S2; FE > 3), and the common characteristic histone modifications of these patterns include the canonical TSS-associated marks H3K4me3, H2AZ, H3K4me1 and H3K9me1 as well as a number of other combinations of histone acetylations, which are known active marks. Examples of several TSS-associated signatures detected by ChAT are shown in Figure 2.

Figure 2A shows the histone modification enrichment profile of the simplest TSS signature, which is characterized by H3K4me3 alone. In Figure 2B, the TSS-associated signature is shown to be enriched with five co-located active histone modifications. Interestingly, a number of bivalent TSS-associated signatures were also found by ChAT. For example, the bivalent signature shown in Figure 2C is characterized by three co-located active marks and a spatially shifted and multi-modal enrichment of the repressive mark H3K27me3. From the perspective of the ChAT algorithm design, the enrichment profiles of the bivalent signature example (Figure 2C) illustrate the ability of the program to find patterns with multiple modes caused by shifted enrichments of different histone modifications.

**Figure 3.**
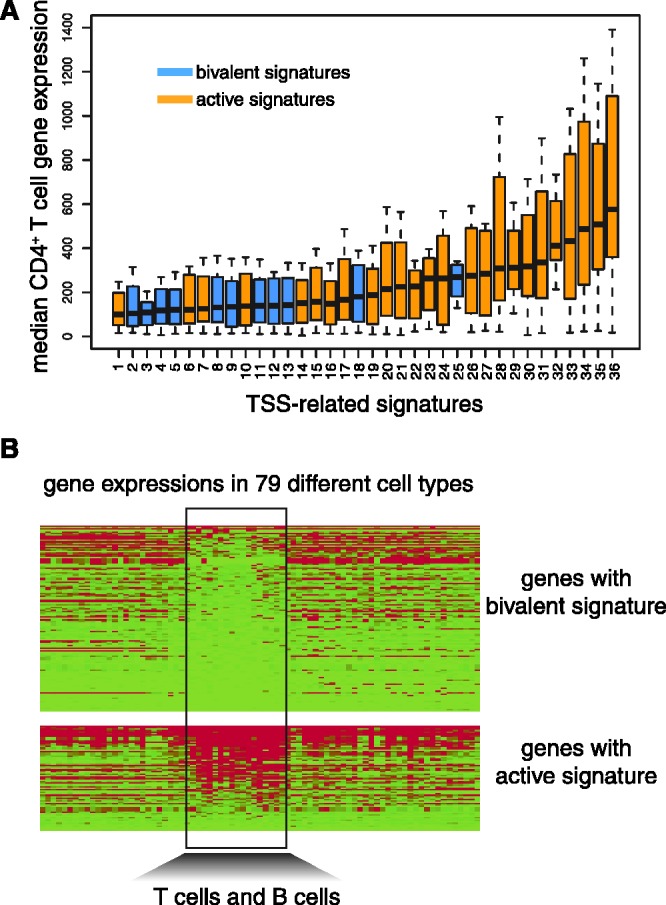
Differential gene expression associated with specific TSS chromatin signatures. (**A**) Median CD4^+^ T-cell expression levels (±1 quartile) of genes with TSS marked by 36 distinct chromatin signatures. Bivalent TSS signatures (blue bars) correspond to lower overall expression levels than active signatures (orange bars). (**B**) Cell-type specific gene expression patterns associated with different TSS chromatin signatures. Gene expression levels across 79 cell types (red, high; green, low) are shown for genes with TSS marked by a bivalent signature versus genes with TSS marked by an active signature. Expression differences are most pronounced for the indicated T cells and B cells.

Analysis of expression levels (35) in CD4^+^ T cells for sets of genes with TSS marked by distinct signatures show that bivalent signatures are associated with lower gene expressions than seen for active signatures (*P* = 4.1 × 10^−^^4^, Mann–Whitney test) (Figure 3A). Furthermore, the lower gene expression levels associated with bivalent signatures, and higher gene expression levels associated with active signatures, are specific to T cells and B cells compared with expression levels in other cell types (Figure 3B). This observation indicates cell-type specific regulatory functions of distinct TSS-associated combinatorial histone modification signatures discovered by ChAT for CD4^+^ T cells.

**Figure 4.**
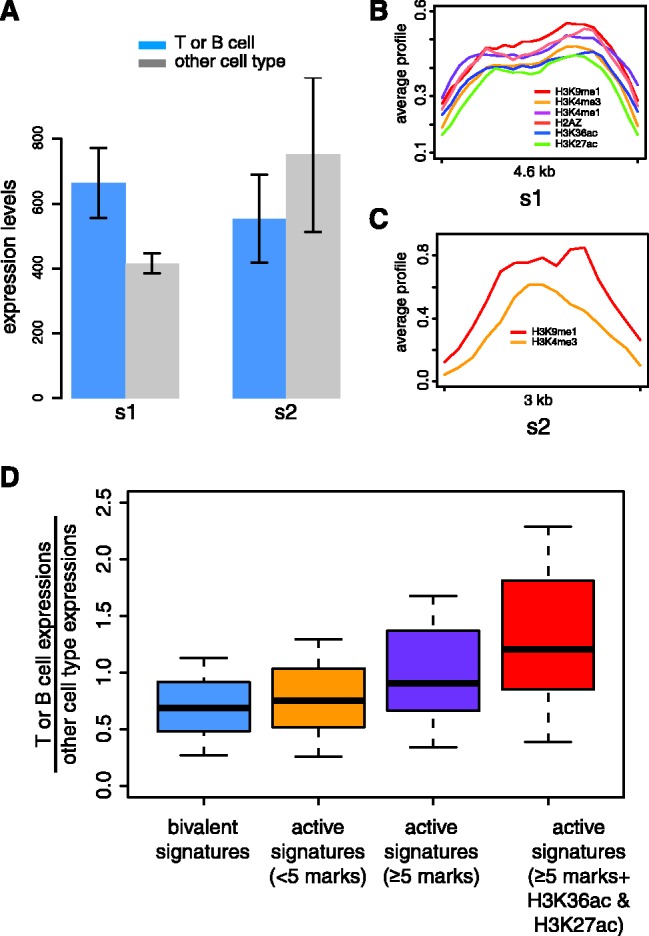
Cell-type specific expression associated with complex chromatin signatures. (**A**) Average (±SD) expression levels (blue, T- or B-cell expression; grey, other cell-type expressions) of genes with TSS marked by two different chromatin signatures (s1 and s2). (**B**) Enrichment profiles showing the average histone modification scores across signature s1. (**C**) Enrichment profiles showing the average histone modification scores across signature s2. (**D**) Box plots showing T- or B-cell specific expression level distributions for different sets of chromatin signatures.

We also observed that sets of genes with similar T- or B-cell expression levels can show very different TSS-associated chromatin signatures. For instance, Figure 4A shows two sets of genes with indistinguishable T- or B-cell expression levels (*P* = 0.7, Mann–Whitney test), but different levels of expression (*P* = 4.9 × 10^−^^3^, Mann–Whitney test) across a panel of numerous other cell-types and tissues (35). In other words, the first set (s1) has a narrower cell-type specific expression pattern, whereas the second set (s2) shows broad expression over numerous cell-types and tissues (Figure 4A). The chromatin signature for the set of cell-type specific genes (s1, Figure 4B) is far more complex, being comprised of six different histone modifications, than the signature made up of two histone modifications seen for the set of broadly expressed genes (s2, Figure 4C). This suggests the possibility that cell-type specific expression is regulated via a more complex chromatin promoter landscape. In fact, when all 36 of the TSS-related chromatin signatures are evaluated, more complex signatures are found to be associated with gene sets that have higher T- or B-cell-type specific expression levels (Figure 4D). The acetylation marks H3K36ac and H3K27ac in particular are associated with high levels of T- or B-cell-type specific expression.

### TTS-associated chromatin signatures

**Figure 5.**
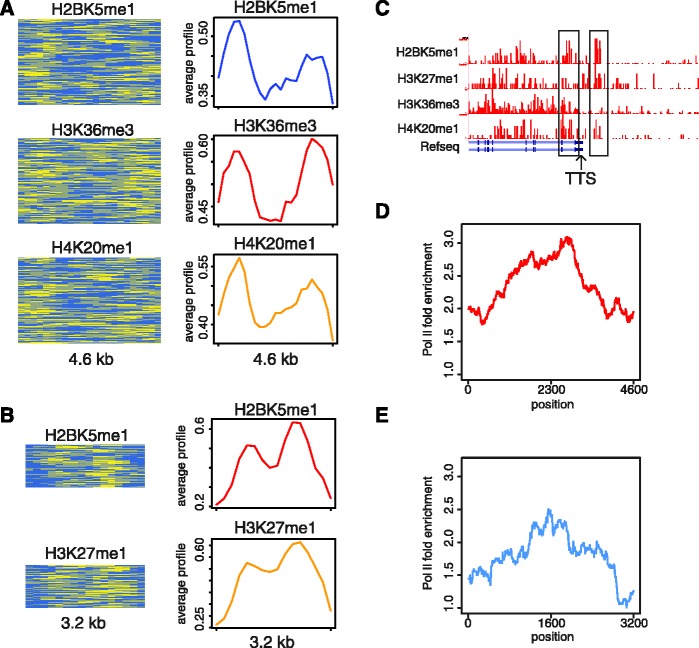
TTS-associated chromatin signatures. TTS signatures associated with three (**A**) and two (**B**) histone modification combinations are shown (histone modification representations described as for Figure 2). (**C**) A specific TTS proximal locus showing adjacent locations of each of these two patterns. (**D**) Pol II enrichment profile within genomic regions marked by the signature shown in (A). (**E**) Pol II enrichment profile within genomic regions marked by the signature shown in (B).

The nature of chromatin signatures around TTS have not been previously characterized as well as those associated with TSS (28,30,36), and this may be due to a lack of coherence in the histone modification patterns found at gene termini. Nevertheless, ChAT was able to discern nine small-sized patterns associated with TTS in CD4^+^ T cells (Supplementary Table S2; FE > 3). The common characteristic marks for these TTS signatures are quite distinct from those seen around TSS and include H2BK5me1, H4K20me1 and H3K27me1. Two examples of TTS-associated signatures are shown in Figure 5A and B. A single genomic region showing adjacent locations of each of these two signatures close to an annotated TTS is shown in Figure 5C. Both of these TTS patterns are bi-modal with two enriched peaks linked by a relatively depleted central region. The relatively low levels of histone modifications seen in the central regions of these patterns may be related to specific protein binding events as has been suggested for the bi-modal patterns of enhancers (15). Consistent with this possibility, these same sets of regions show peaks of RNA polymerase II (Pol II) binding that corresponds to the locations of the depleted regions in the bi-modal patterns (Figure 5D and E). With respect to the ChAT algorithm design, the bi-modal patterns seen at TTS point to the utility of gaps in the chromatin profile alignments, which allow chromatin patterns to extend beyond variant regions and include multiple peaks of individual histone modifications.

### Enhancer-associated chromatin signatures

**Figure 6.**
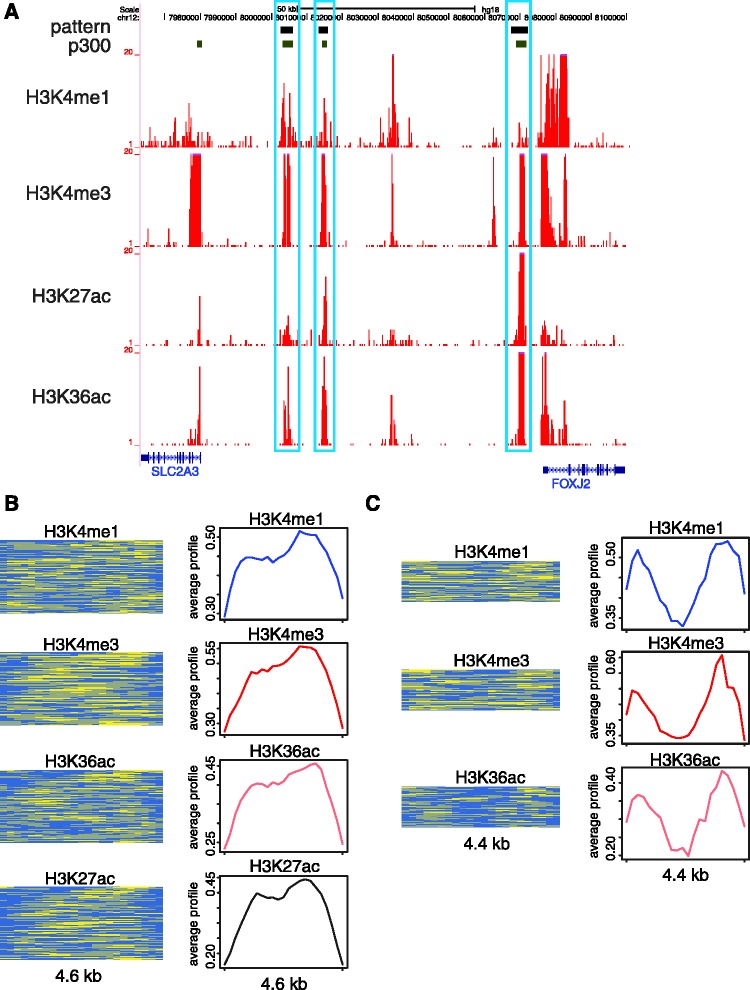
Enhancer-associated chromatin signatures. (**A**) ∼100 kb genomic region with three locations (black bars) marked by a specific enhancer-associated signature composed of co-located peaks of H3K4me1, H3K4me3, H3K27ac and H3K36ac (ChIP-seq tag counts in red). All of the three locations overlap with p300 binding sites. (**B**) Histone modification enrichment profiles of an enhancer-associated mono-modal signature. (**C**) Enrichment profiles of an enhancer-associated bi-modal signature. Histone modification representations are as described for Figure 2.

Chromatin signatures characteristic of enhancers have been characterized in a number of studies (6,7,12,15,24–26), many of which rely on the positions of p300 binding sites to identify enhancer locations. We also took the locations of p300 binding sites (33) to indicate putative enhancers and found that ChAT characterized 18 small-sized signatures that are co-located with these sites (Supplementary Table S2; FE > 3). The common characteristic marks of these patterns include the canonical enhancer-associated marks H3K4me1 and H3K4me3 along several other histone acetylations (Figure 6A). Examples of enhancer-associated signatures detected by ChAT are shown in Figure 6B and C; these two distinct signatures are characterized by similar sets of histone modifications with markedly different profile shapes, i.e. mono-modal (Figure 6B) versus bi-modal (Figure 6C). The different shapes of this kind discovered by ChAT may point to distinct dynamics of histone-modifying enzymes and/or DNA binding proteins between the two sets of enhancers, indicative of the utility of the algorithm for discovering specific chromatin-based regulatory mechanisms.

### Conserved non-coding element-associated chromatin signatures

**Figure 7.**
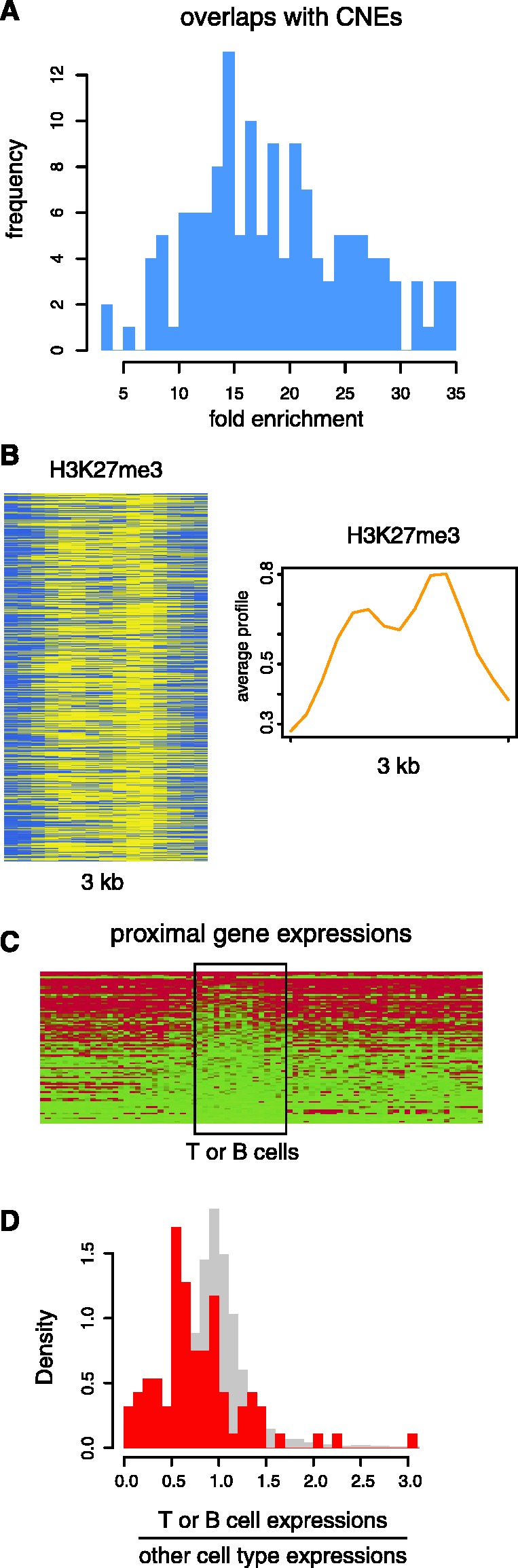
CNE-associated chromatin signatures. (**A**) Distribution of FEs of CNEs for all small-sized signatures. (**B**) Histone modification enrichment profiles (as described for Figure 2) for a repressive signature highly enriched within CNEs. (**C**) Cell-type specific expression levels for genes proximal to CNEs bearing the repressive signature shown in (B). (**D**) Distribution of the ratios of T- or B-cell average expressions and other cell type average expressions for genes shown in (C) (observed, red; expected, grey). Observed ratios are significantly smaller than expected ratios calculated from gene expression levels randomly simulated across cell-types and tissues (*P* = 1.3 × 10^−10^, Mann–Whitney test).

Conserved non-coding elements (CNEs) are non protein-coding sequences that have been found to be anomalously conserved between species; CNEs are of interest because they are thought to correspond to regulatory regions that have been conserved by purifying selection based on their functional utility (37). We evaluated CNEs characterized via the comparison of genome sequences from 28 vertebrate species for the presence of chromatin signatures discovered with the ChAT algorithm and found that all 144 signatures show substantial overlap (FE > 3) with the CNEs (Figure 7A and Supplementary Table S2). This result is consistent with the presumed regulatory activity of CNEs. Not surprisingly, most of the CNE-associated signatures are made up of active histone marks and tend to be associated with TSS or enhancers; such CNEs are likely to be active regulatory elements in CD4^+^ T cells. However, a number of CNEs were also found to be associated with repressive chromatin signatures. For example, a simple chromatin signature made up of the repressive mark H3K27me3 (Figure 7B) is highly enriched over CNEs (FE = 18.4). We surmised that these CNEs may represent regulatory elements that are active in other cell-types but repressed in a specific manner in T or B cells. To evaluate this possibility, we checked the expression levels of the genes most proximal to these CNEs for their expression across 79 human tissues and cell-types (35). These genes do appear to be repressed in T or B cells in a cell-type specific manner, because they are expressed at higher levels across other cell types compared with T or B cells (Figure 7C and D).

### Bivalent chromatin signatures associated with L1 retrotransposons

**Figure 8.**
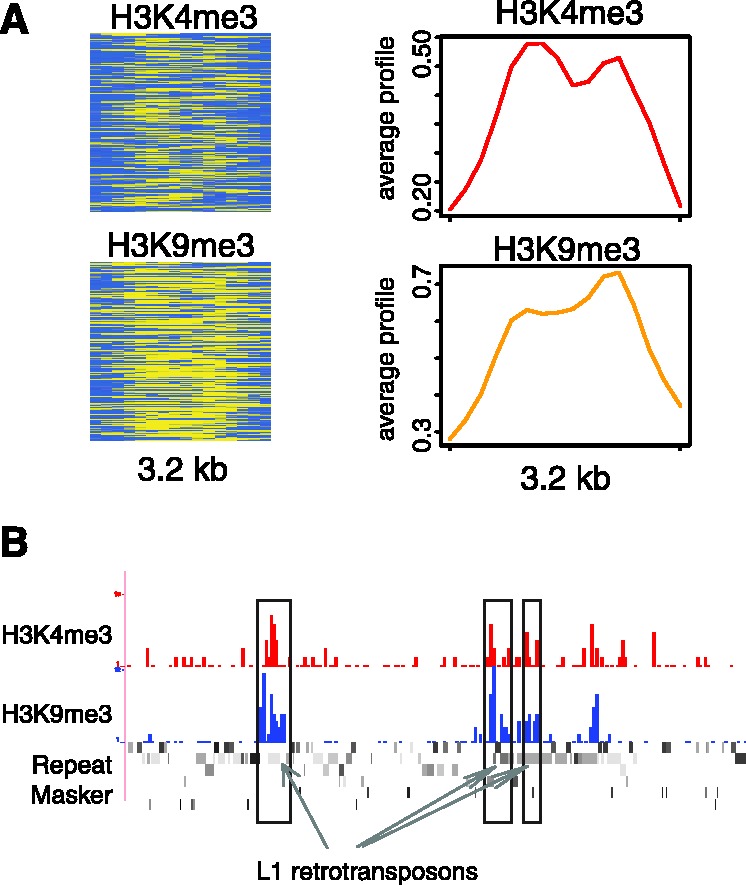
A bivalent chromatin signature associated with L1 retrotransposons. (**A**) Histone modification enrichment profiles (as described for Figure 2) for the bivalent signature. (**B**) A single genomic region with three locations marked by the L1 characteristic bivalent signature. ChIP-seq tag counts are shown for the active mark H3K4me3 (red) and the repressive mark H3K9me3 (blue).

Bivalent chromatin signatures, composed of co-located active and repressive histone modifications (38,39), have previously been associated with TSS sequences, and the ChAT algorithm was also able to detect such bivalent signatures at TSS in CD4^+^ T cells (Figures 2C and 3). Application of ChAT here revealed two bivalent signatures that were not found to be associated with TSS: H3K9me3 and H3K36me3 (Supplementary Figure S2) along with H3K4me3 and H3K9me3 (Figure 8A). Interestingly, both of these bivalent signatures were found to be highly enriched within L1 retrotransposon sequences; 68.4% of the genomic regions marked by the H3K9me3-H3K36me3 signature overlap with L1 as do 77.0% of genomic regions marked by H3K4me3 and H3K9me3. A broad genomic region with several L1 encoded segments that overlap the H3K4me3-H3K9me3 signatures can be seen in Figure 8B.

This particular bivalent pattern has previously been associated with imprinted genomic loci wherein genes tend to be expressed in a mono-allelic fashion based on the parent of origin for the allele (39). Interestingly, a number of studies have also shown that L1 retrotransposons are enriched in-and-around imprinted genomic loci (40–43). Thus, the enrichment of these bivalent signatures on L1 retrotransposons may point to a chromatin-based mechanism by which L1 sequences contribute to the mono-allelic expression of human genes. On the other hand, such bivalent patterns may actually result from ChIP-seq analyses performed heterogeneous cell populations with the locations in some cells marked by active modifications and others with repressive modifications. In this case, the patterns revealed by the algorithm would represent an artifact of the ChIP-seq experimental design.

### Large-sized chromatin signatures

**Figure 9.**
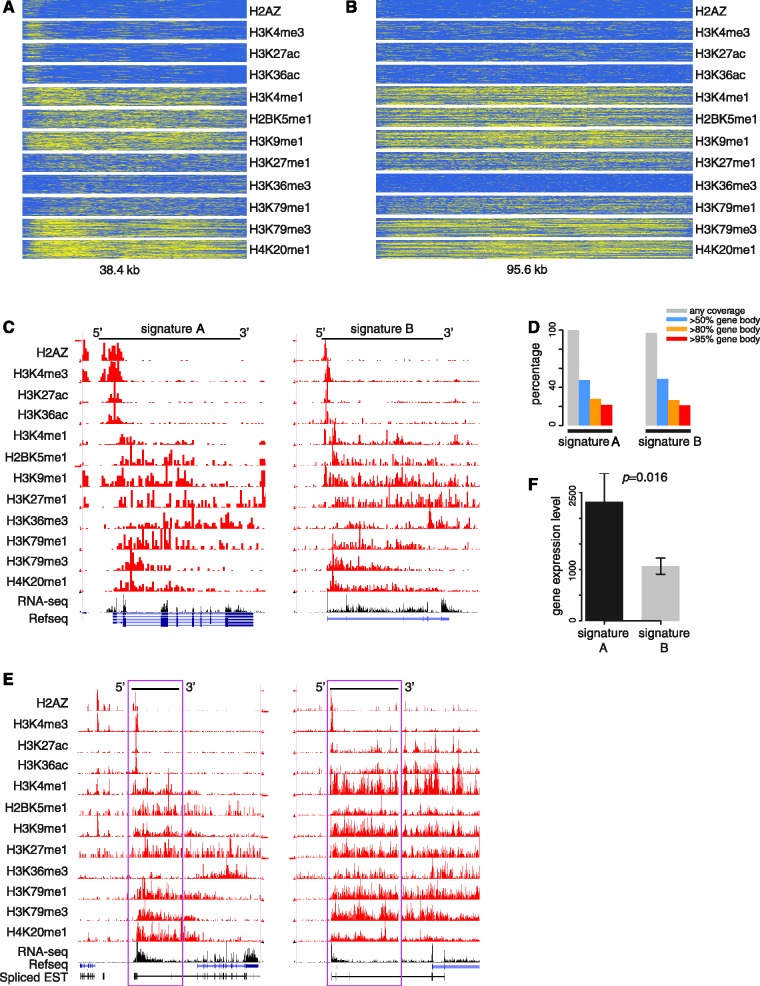
Large-sized chromatin signatures associated with gene bodies. (**A**, **B**) Histone modification enrichment profiles (as described for Figure 2) are shown for two chromatin signatures composed of the same constituent modifications and spatial patterns with distinct sizes. (**C**) Specific instances of each signature co-located with human gene bodies are shown with modification ChIP-seq tag counts in red and RNA-seq tag counts in black. (**D**) Percentage of these two large-sized signatures that overlapping with gene bodies (grey, any coverage; blue >50% coverage; orange >80% coverage; red >95% coverage of the gene body). (**E**) Two examples where signature B is co-located with individual genomic regions that are annotated as intergenic but show evidence of being genic from RNA-seq and spliced EST data. (**F**) Average CD4^+^ T-cell expression levels for genes marked by signatures A and B.

The ChAT algorithm places no restriction on the size of chromatin signatures that it can identify, and we found 27 large-sized signatures in CD4^+^ T cells ranging from 10 to 100 kb in length. These large-sized chromatin signatures can be classified into two groups. The first group contains long contiguous co-located blocks of repressive marks, presumably representing heterochromatic or repressive chromatin domains. The second group shows more complex and potentially interesting patterns resembling the known H3K4me3-H3K36me3 domains, which are associated with gene bodies and long non-coding RNAs (3,5,44). For example, the signatures shown in Figure 9A and B (see also Supplementary Figures S3 and S4) are characterized by the presence of similar active marks albeit over different size ranges. In both cases, the long chromatin signatures show punctate enrichments of several active marks at one end of the pattern together with broader enrichments of different active marks throughout the rest of the signature. These two large-sized signatures show substantial overlaps with gene bodies (Figure 9C), suggesting the utility of ChAT for annotating genes.

However, while more than 90% of these two large-sized signatures do overlap with known gene bodies (Figure 9D), there is still a small fraction which does not overlap with gene bodies. For example, Figure 9E shows two specific genomic regions where the signatures do not overlap with annotated gene models. Inspection of RNA-seq and spliced EST data from these regions suggests the possibility that the regions marked by these chromatin signatures represent as yet uncharacterized alternative promoters of nearby genes.

The biggest difference in the enrichment levels for any individual mark between these two patterns is seen for H3K36me3, a mark of transcriptional elongation (3,15). Consistent with this observation, genes marked by these two chromatin signatures show different expression levels in CD4^+^ T cells (*P* = 0.016; Figure 9F). These data underscore the functional relevance of slight differences in chromatin signatures that are able to be distinguished by the ChAT algorithm.

**Figure 10.**
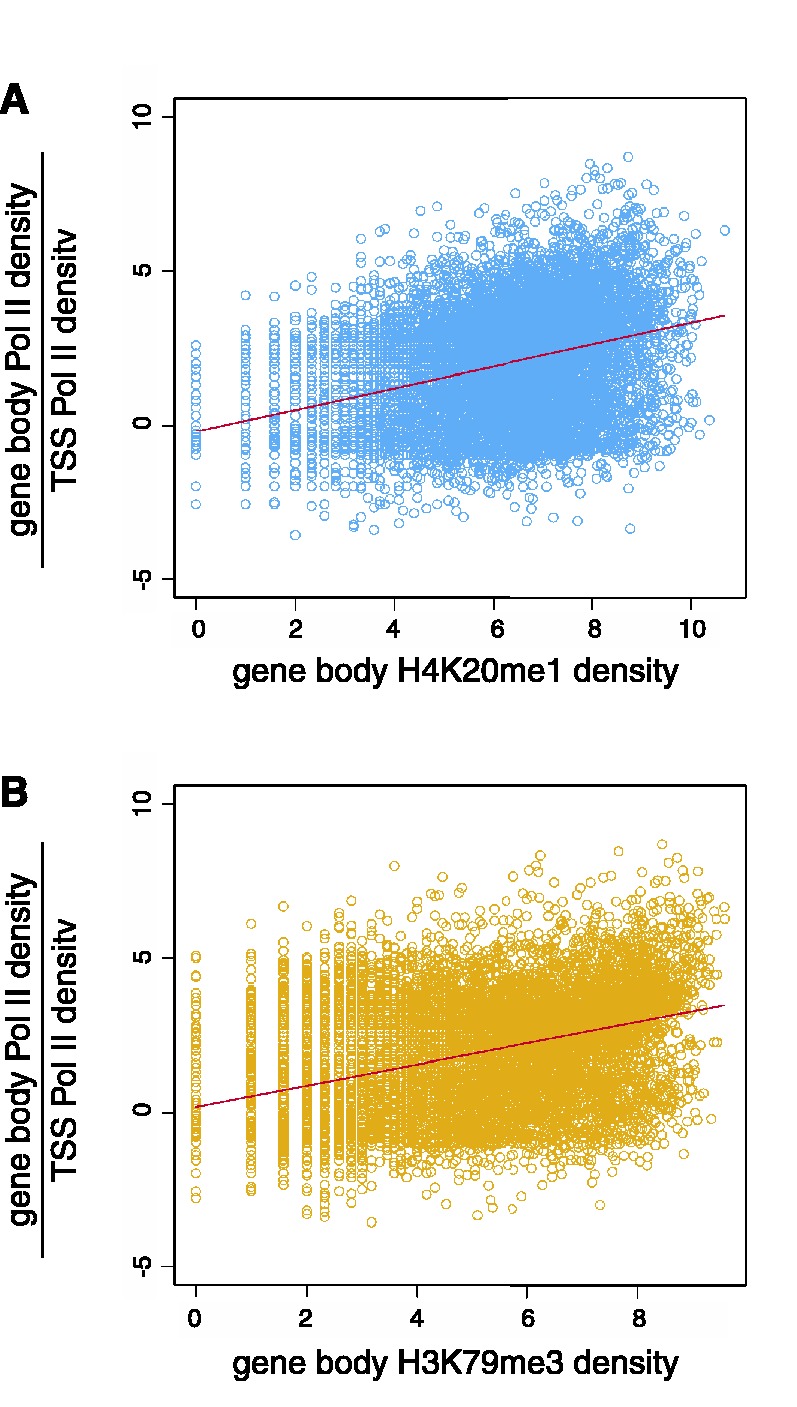
Transcriptional pause release associated with H4K20me1 and H3K79me3. The ratio of Pol II density downstream of TSS (+1 to +5 kb) over its density around TSS (−1 to +1 kb) is positively correlated with the density of downstream H4K20me1 (**A**, Spearman’s *ρ* = 0.54) and H3K79me3 (**B**, Spearman’s *ρ* = 0.51).

Both of these long chromatin signatures show enrichment of H4K20me1 and H3K79me3 that tend to be located within gene bodies and start just downstream of TSS (Figure 9A–C). This suggests the possibility that these marks are associated with transcriptional pause release, a phenomenon whereby Pol II complexes paused at promoter regions are allowed to proceed into gene bodies to facilitate active transcription of the genes (45,46). Previously, the relative levels of bound Pol II seen in promoter proximal versus downstream regions have been used to evaluate the extent of transcriptional pause release (47,48). Here, we show that the ratio of gene body-to-TSS Pol II density is positively correlated with the gene body levels of H4K20me1 (Figure 10A) and H3K79me3 (Figure 10B) consistent with a role for these marks in transcriptional pause release.

The discoveries of those complex large-sized signatures highlight the performance of ChAT with respect to several aspects of the algorithm design. First of all, the large-size of these signatures underscores the advantage of predicting chromatin signatures without size restrictions. Second, the prediction of large-sized signatures was facilitated by the ability of the algorithm to extend histone modification profile alignments through the use of gaps in the dynamic programming implementation. Third, the complex histone modification enrichment profiles apparent in these signatures, i.e. the specific enrichments of several histone modifications over a narrow range of the pattern and the broad enrichments of other marks in the rest of the pattern, demonstrates the ability of the algorithm to detect patterns with spatially shifted multi-modal enrichments of multiple modifications.

## CONCLUSIONS

We developed ChAT an unsupervised algorithm for the discovery and characterization of recurrent combinatorial histone modification patterns, i.e. chromatin signatures. ChAT utilizes a novel dynamic programming and hierarchical clustering approach to relate and group similar chromatin signatures dispersed across the genome. The algorithm was explicitly designed to provide complementary utility with respect to existing methods. For example, ChAT can identify chromatin signatures across a vast range of different sizes, it finds multi-modal chromatin signatures composed of individual histone modifications that are spatially shifted as well as complex signatures composed of conserved and variant segments, and ChAT can also distinguish between chromatin signatures that are made up of the same constituent histone modifications with different shapes. The algorithm also employs an explicit statistical criterion that provides confidence levels for the grouping of similar chromatin signatures.

We applied ChAT to the analysis of genome-wide histone modification maps in human CD4^+^ T cells. The algorithm was able to discern combinatorial histone modification patterns previously observed to be associated with genomic regulatory features such as TSS and enhancers, serving as a proof of its utility for the discovery of functionally relevant chromatin signatures. Perhaps more interestingly, we were also able to discover a number of previously unknown chromatin signatures with ChAT. For example, we discovered novel chromatin signatures associated with TTS, enhancers and CNEs. We were also able to uncover functional associations, based on enrichment of chromatin signatures at specific genomic regulatory features, which point to novel chromatin-based mechanisms of gene regulation. For example, we found evidence for the role of complex chromatin signatures, made up of numerous co-located histone modifications, in the cell-type specific regulation of human genes. We also found evidence suggesting that L1 retrotransposons can influence the mono-allelic expression of human genes by creating a local genomic environment enriched for specific bivalent chromatin signatures. Finally, novel long chromatin signatures found to be associated with human genes suggest a role for the H4K20me1 and H3K79me3 histone modifications in transcriptional pause release. The discovery of these novel chromatin signatures and functional associations underscores the potential utility of the algorithm to provide novel biological insight and to help focus future experimental efforts for the characterization of chromatin-based regulatory mechanisms.

## SUPPLEMENTARY DATA

Supplementary Data are available on NAR Online: Supplementary Tables 1 and 2, Supplementary Figures 1–4 and Supplementary File 1.

## FUNDING

Alfred P. Sloan Research Fellowship in Computational and Evolutionary Molecular Biology [BR-4839 to J.W. and I.K.J.]; Georgia Tech Integrative BioSystems Institute pilot program grant (to J.W. and I.K.J.); the Buck Institute Trust Fund (to V.V.L.). Funding for open access charge: School of Biology, Georgia Institute of Technology.

*Conflict of interest statement*. None declared.

## Supplementary Material

Supplementary Data
