# Supplementary material for: Chromatin signature discovery via histone modification profile alignments
Source: Nucleic Acids Res. 2012 Sep 18;40(21):10642–56. doi: 10.1093/nar/gks848 (PMC3505981; doi:10.1093/nar/gks848)
Supplement: Supplementary Data [file supp_40_21_10642__index.html]

Chromatin signature discovery via histone modification profile alignments — Chromatin signature discovery via histone modification profile alignments — Supplementary Data 

# Chromatin signature discovery via histone modification profile alignments

## Supplementary Data

files

**Files in this Data Supplement:**

- Supplementary Data - pdf file
