# Supplementary material for: Chromatin signature discovery via histone modification profile alignments
Source: Nucleic Acids Res. 2012 Sep 18;40(21):10642–56. doi: 10.1093/nar/gks848 (PMC3505981; doi:10.1093/nar/gks848)

Supplementary Information for:

Chromatin signature discovery via histone modification profile alignments

Jianrong Wang, Victoria V. Lunyak and I. King Jordan

Contents

Instructions for installing and running the ChAT software 2 - 4

Supplementary Table S1 5

Supplementary Figure S1 6

Supplementary Table S2 7

Supplementary Figure S2 8

Supplementary Figure S3 9

Supplementary Figure S4 10

Instructions for installing and running the ChAT software

1. Preparation:

In order to run ChAT, you need to:

1) Download the compressed folder "ChAT_package.tar.gz" from http://jordan.biology.gatech.edu/page/software/ChAT;
2) Decompress the folder. There are three files within the created folder: A) ChAT, B) clustering.R and C) cluster_figure.R.
3) Make sure these files are always kept in the same folder.
4) Make sure R program is already installed on your computer.
5) Check the shebang line of the ChAT file and correct it by the path of *env* of your computer.
6) Add the directory of the folder ChAT_package into the PATH.

2. Command-line of ChAT:

The command line for ChAT is:

$ ChAT -i [the directory where the set of input files located] -o [the output directory where both the final and intermediate files located] -m [the file of the list of histone modifications] -d [the file of the list of critical histone modifications used for initial grouping] -c [the file of the list of chromosomes] -p [*p*-value threshold to cut the hierarchical tree] -b [bin size]

The detailed explanations of the parameters can be found in Section 4.

One example of running ChAT is:

$ ChAT -i /home/CD4_sample_data -o sample_pattern -m mark_name.txt -d critical_mark.txt -c chromosome.txt -p 0.05 -b 200

This command takes the Wiggle format histone modification files (must be named as *.wig) located in "/home/CD4_sample_data" folder as the inputs and create the directory "sample_pattern" to store all the final and intermediate results. "mark_name.txt" contains the list of histone modifications (each row has a histone modification name) that are consistent with the file names in the input directory. "critical_mark.txt" contains a subset of histone modifications used for initial grouping. "chromosome.txt" contains a list of chromosomes (each row has a chromosome name that are consistent with the chromosome names in the wiggle format input files) under consideration. The p-value threshold used to cut the hierarchical tree is set as 0.05. The bin size is set as 200bp using "-b".

3. File Format:

(A) Input files

Corresponding to each individual histone modification, there is a Wiggle format file of the ChIP-seq data. All of the files need to be named as “histone_mark_name.wig”. For example, “H3K36me3.wig” for H3K36me3. All the files must be stored in the same directory. The name of the directory is the most important parameter for ChAT.

A file of the list of all the histone modifications under consideration need to be provided. Each row has the name of a histone modification.

A file of the list of critical histone modifications for initial grouping need to be provided. Those modifications are important marks based on a priori biological knowledge. This list must be a subset of the modifications under consideration. Each row has the name of a critical histone modification.

A file of the list of all the chromosomes under consideration need to be provided. Each row has the name of a chromosome. The names need to be consistent with the chromosome names in the input wiggle format files.

(B) Output files

All the output files are stored in the created folder specified by “-o”. The most important final results are saved in 2 folders.

The BED format tracks of genomic locations sharing specific combinatorial chromatin signatures are stored in “BED_tracks”.

The average histone modification profiles of each signature and the corresponding enrichment curves in PDF files are stored in “Signature_info”.

4. Parameters:

-i: The directory where all the wiggle format input files (one file for each histone modification) are located. The wiggle format files must be names as “*.wig”.

-o: The output directory where all the final and intermediate results are stored.

-m: The file with the list of histone modifications under consideration. Each row has the name of one histone modification. They need to be consistent with the name of the wiggle format input files.

-d: The file with the list of critical histone modifications used for initial grouping. Each row has the name of one histone modification.

-c: The file with the list of chromosomes under consideration. Each row has the name of one chromosome. They need to be consistent with the chromosome names in the wiggle format input files.

-p: The *p*-value threshold used to cut the hierarchical tree, default value: 0.05.

-b: The size of bin, default value 200 (bp).

-h,-help: Display brief explanations of parameters.

5. Computational performance:

ChAT is tested on a Ubuntu Linux server (with memory 8 Gb) to identify combinatorial signatures based on the ChIP-seq datasets of 14 histone methylations on human chromosome 2, and it takes 25.5 minutes to produce the combinatorial chromatin signatures.

Supplementary Table S1: Overview of the algorithmic features of ChAT and related software.

	ChromaSig	CoSBI	ChromHMM	Segway	ChAT
Free of size-restriction			X	X	X
No use of motif seeds		X	X	X	X
Multi-modal signatures			X	X	X
Deal with flexible histone modification distributions			X	X	X
Classify distinct shapes					X
Intrinsic statistical criterion	X				X

Supplementary Figure S1: Algorithm performance comparison. A set of histone methylation ChIP-seq datasets on human chromosome 2 are used to test four specific algorithmic features of ChAT, ChromaSig and CoSBI. The three softwares identify similar chromatin signatures for a standard mono-modal pattern (A). For a bi-modal pattern identified by ChAT, ChromaSig and CoSBI only found mono-modal signatures (B). For a set of genomic locations enriched with the same set of histone modifications, ChAT discriminate two patterns with distinct enrichment shapes (C). ChAT identified a complex large-sized signature (~82 kb) for a set of genomic locations, while ChromaSig found a number of small-sized signatures as parts of the large signature (D).

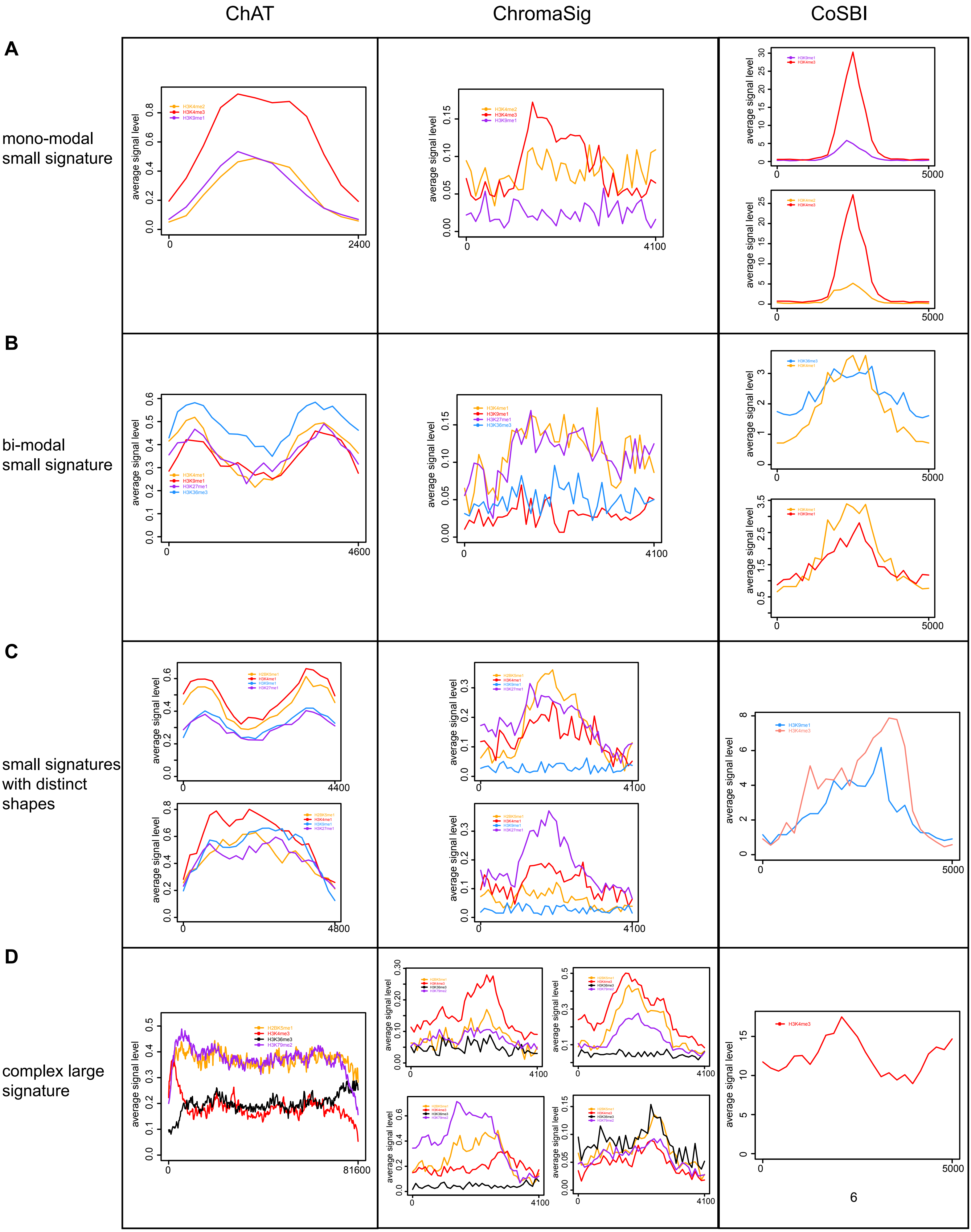

Supplementary Table S2: Enrichments of small-sized combinatorial histone modification patterns with functional genomic features. The numbers and fractions of combinatorial histone modification patterns enriched with specific genomic features are summarized.

Genomic Features	No. patterns enriched with FE[a]>3	No. patterns enriched with FE>5	No. patterns enriched with FE>8
TSS[b]	36 (25.0%)	21 (14.6%)	8 (5.6%)
TTS[c]	9 (6.3%)	0	0
p300[d]	18 (12.5%)	16 (11.1%)	12 (8.3%)
DNase I[e]	60 (41.7%)	51 (35.4%)	40 (27.8%)
CNE[f]	144 (100.0%)	142 (98.6%)	137 (95.1%)

[a]FE: ratios of the fractions of patterns overlapping with the specific features over the genomic fractions of the corresponding features.
[b]TSS: transcription start site.
[c]TTS: transcription termination site.
[d]p300: binding sites of p300.
[e]DNase I: DNase I hypersensitive sites.
[f]CNE: Conserved non-coding elements predicted based on sequence alignments of 28 vertebrate species (data downloaded from UCSC genome browser).

Supplementary Figure S2: Histone modification profiles for H3K36me3-H3K9me3 bivalent pattern. Genomic locations with this specific bivalent pattern are aligned and levels of H3K36me3 and H3K9me3 are shown as heatmaps on the left (yellow - higher levels, blue - lower levels). The average profiles of histone modifications of this pattern are shown on the right.

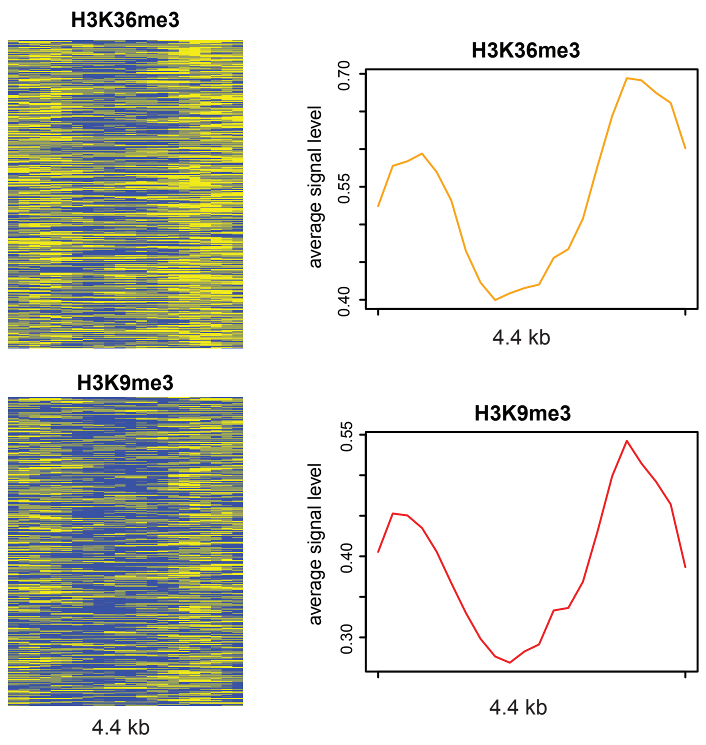

Supplementary Figure S3: Average histone modification profiles for the large pattern example A. Each curve shows the average profile of a specific histone modification of genomic locations with the same pattern.

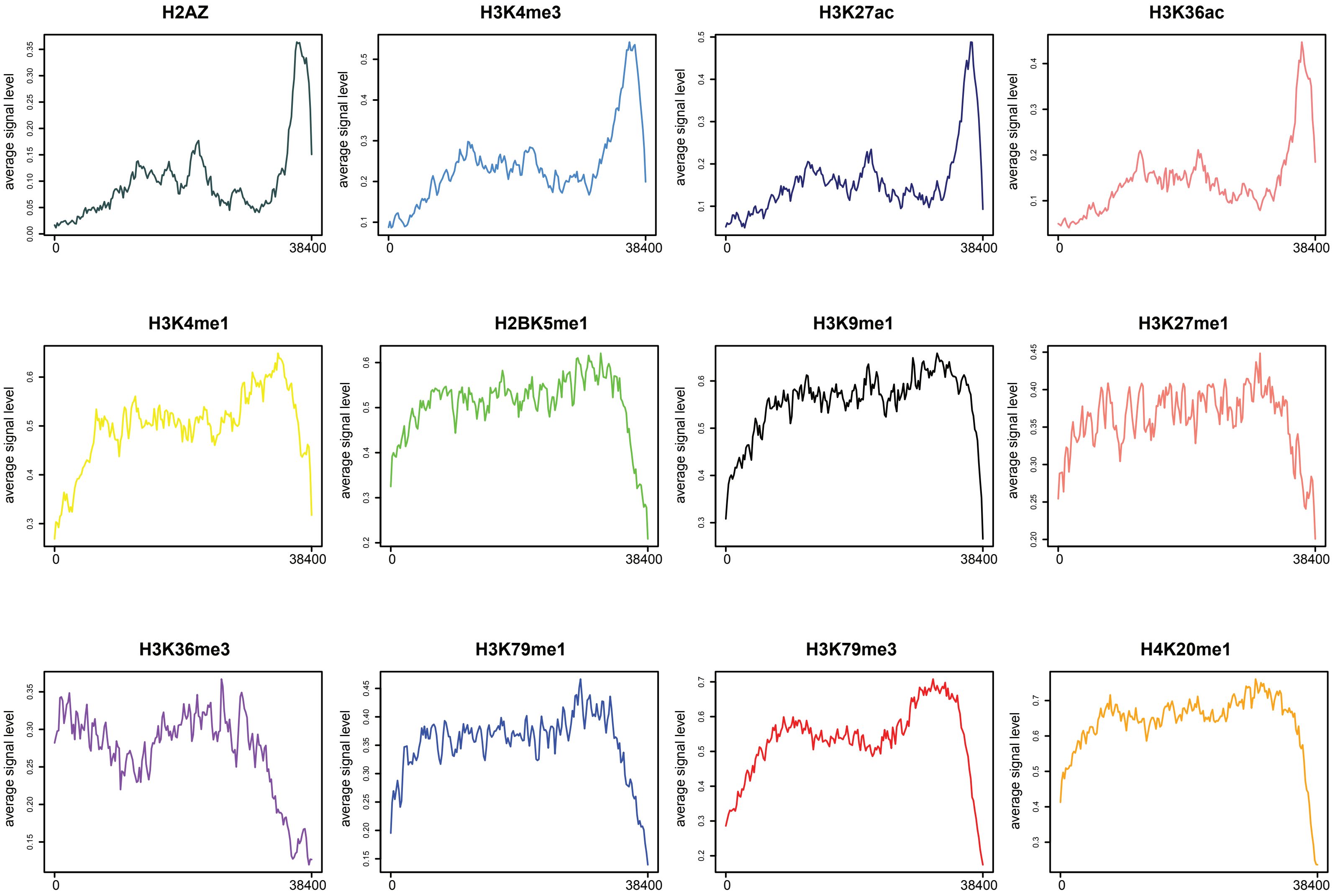

Supplementary Figure S4: Average histone modification profiles for the large pattern example B. Each curve shows the average profile of a specific histone modification of genomic locations with the same pattern.

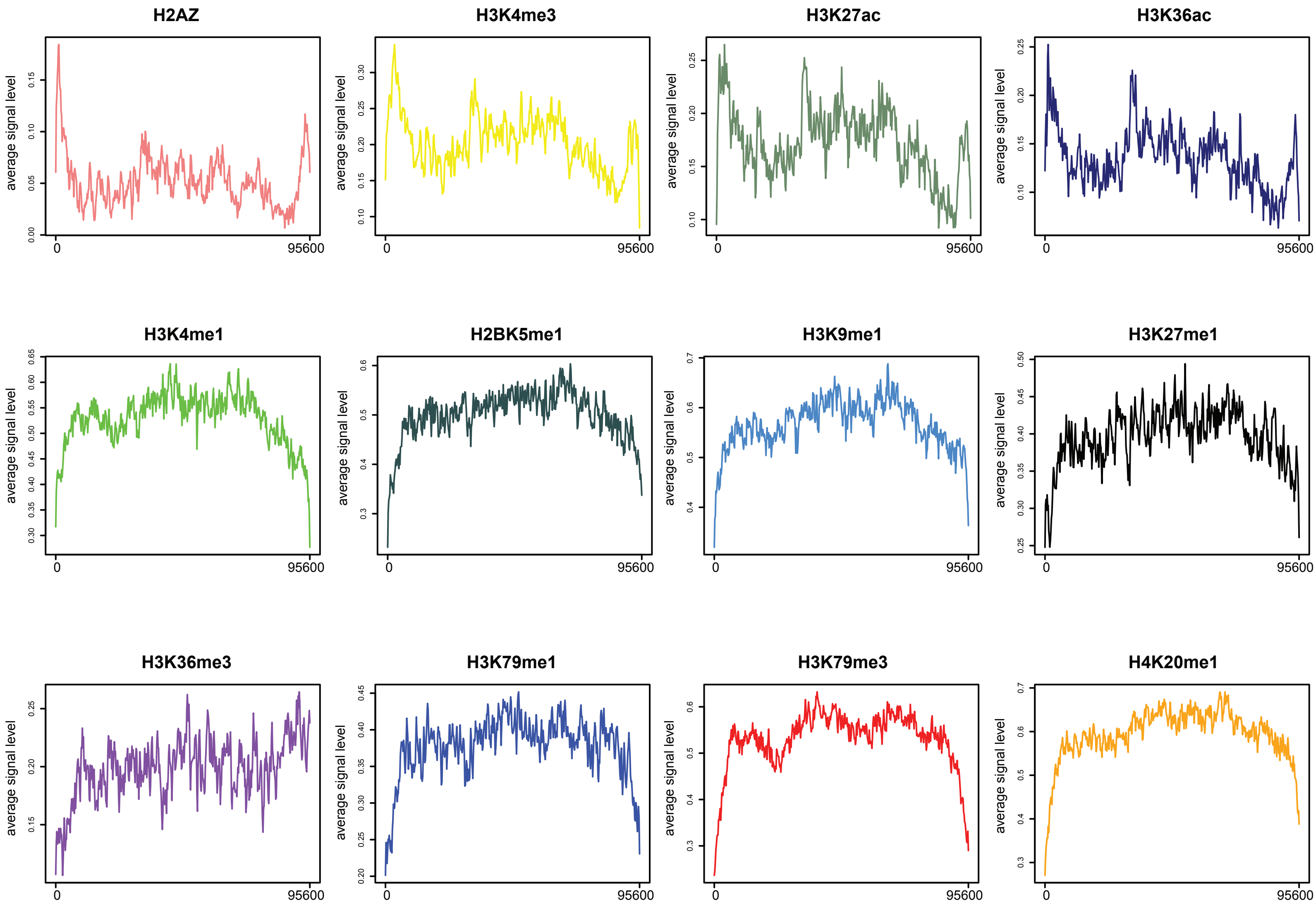

Supplement: Supplementary Data [file supp_gks848_nar-01258-n-2012-File012.pdf]
